# Innovative Hyperbranched Polybenzoxazine-Based Graphene Oxide—Poly(amidoamines) Nanomaterials

**DOI:** 10.3390/polym12102424

**Published:** 2020-10-21

**Authors:** Elena Iuliana Bîru, Sorina Alexandra Gârea, Horia Iovu

**Affiliations:** 1 Advanced Polymer Materials Group, University Politehnica of Bucharest, Gh. Polizu Street, 011061 Bucharest, Romania; iuliana.biru@upb.ro (E.I.B.); sorina.garea@upb.ro (S.A.G.); 2 Academy of Romanian Scientists, 54 Splaiul Independentei Street, 050094 Bucharest, Romania

**Keywords:** benzoxazine functionalization, dendrimers, polymerization

## Abstract

The covalent functionalization of graphene oxide (GO) surface with hyperbranched benzoxazine (BZ) structures has been achieved using poly(amidoamine) dendrimers (PAMAM) of different generations. By increasing the PAMAM generation, multiple benzoxazine rings were synthesized decorating the GO layers. The polymerization process and the exfoliation behavior were investigated. The novel BZ-functionalized GO hybrid materials were characterized by a combination of techniques such as FT-IR, XPS, and ^1^H-NMR for the confirmation of benzoxazine formation onto the GO layer surfaces. Raman and XRD investigation showed that the GO stacking layers are highly disintegrated upon functionalization with hyperbranched benzoxazine monomers, the exfoliation being more probably to occur when lower PAMAM generation (G) is involved for the synthesis of hybrid GO-BZ nanocomposites. The polymerization of BZ rings may occur either between the BZ units from the same dendrimer molecule or between BZ units from different dendrimer molecules, thus influencing the intercalation/exfoliation of GO. DSC data showed that the polymerization temperature strongly depends on the PAMAM generation and a significant decrease of this value occurred for PAMAM of higher generation, the polymerization temperature being reduced with ~10 °C in case of GO-PAMAM(G2)-BZ. Moreover, the nanoindentation measurements showed significant mechanical properties improvement in case of GO-PAMAM(G2)-BZ comparing to GO-PAMAM(G0)-BZ in terms of Young modulus (from 0.536 GPa to 1.418 GPa) and stiffness (from 3617 N/m to 9621 N/m).

## 1. Introduction

Nowadays the synthesis of versatile polymers represents an emerging demand for industrial sectors that require easy to handle, yet high-performance polymers for construction materials, electronic devices, or aerospace industry. Polybenzoxazines (PBZs) are a unique class of thermoset resins that in many cases surpass the phenolic and epoxy resin properties in terms of thermal resistance, UV and chemical stability, water absorption, void formation, and volumetric shrinkage during the curing process [1,2,3]. These remarkable features of PBZs are generated by the rich molecular flexibility of the benzoxazine monomers that allows the synthesis of complex polymerizable structures for tailored properties [4].

Benzoxazine (BZ) chemistry is based on the Mannich condensation reaction between phenols, formaldehyde, and primary amines with the formation of a six-membered oxazine ring attached to benzene structure [5]. Therefore, distinctive BZ monomers may be obtained for different purposes by solely using suitable functionalized reagents. PBZ resins are produced via further thermal ring-opening polymerization (ROP) forming numerous –OH groups that are inter- and intramolecularly hydrogen bonded [6,7]. Although PBZs exhibit high glass temperature (Tg ≅ 170–340 °C), high char yield and better flammability resistance compared to conventional thermoset resins [1], the curing process takes place at relatively high temperatures (160–220 °C) restraining the usage of these polymers at industrial scale [8]. Thus, several strategies were developed in order to reduce the polymerization temperature by designing monomers with self-catalytic effect on the ROP as the –COOH and –OH groups showed effective contribution to accelerate the process [5,9,10,11,12]. However, the most significant results were obtained when extra-catalysts were employed such as cyanuric chloride [13], amines [14], sulfonates [15], lithium [16], and amine salts [17,18] that not only lower the ROP temperature but contribute to prolongation of the product life storage also. Recently, studies showed that incorporation of graphene oxide (GO) into polymer-based composite formulations significantly contribute to reduction of polymerization temperature [19]. GO represents a monolayer of carbon atoms covalently bonded in sp^2^/sp^3^ hybridization decorated with oxidized organic species. Being a graphene derivative, GO exhibits large surface area, great mechanical properties [20], thermal conductivity and good optical transmittance. Due to its many reactive oxygen functionalities such as epoxy, hydroxyl, and carboxyl [21], GO represents a fruitful platform for polymer composites with applications for sensors [22,23,24], electronics [25,26], corrosion protection [27], or environmental issues [28]. Zeng and co-workers studied the curing behavior of bisphenol A-based benzoxazine in the presence of GO [29] showing that the polymerization temperature is decreased with ca. 25 °C when 3 wt% GO is incorporated. Moreover, the authors investigated the effect of carboxylated GO (GO-COOH) on the curing process of the BZ monomer demonstrating not only the reduction of the polymerization temperature, but also the crosslinking density is significantly enhanced, improving the Tg and thermal properties of the final nanocomposite [30]. Nevertheless, the mechanical properties are increased reducing the brittleness of the pure PBZ. However, it is well known that graphene derivatives exhibit tendency to agglomerate due to π-π * stacking interactions and van der Waals attractions between layers, hindering the inclusion of molecules [31]. Thus, the exfoliation degree of GO layers highly influences the final properties of the materials. For better dispersion of GO into PBZ matrix, high degree of functionalization of its surface with BZ groups is required. In this regard, Ishida and his group proposed the introduction of BZ functionalities onto the GO surface by covalently attaching the BZ monomer separately synthetized through click chemistry route [32]. The synthesis of the BZ rings grafted from the surface of the GO layers is sparsely reported in the literature. We have previously reported a new approach for producing the PBZ/GO nanocomposites employing GO [33] and aminated–GO [34] as starting reagents for BZ synthesis. By this method, the novel hybrid BZ/GO monomers were polymerized showing that the ROP may take place either between the BZ groups located on the same GO layer (in-graphene polymerization) or between the BZ groups located on different GO layers (out-graphene polymerization) and that the ratio between these two types of polymerization strongly influence the exfoliation of GO layers within the PBZ matrix. Herein the synthesis of new GO–hyperbranched BZ structures is reported in order to produce higher content of BZ rings directly onto the GO surface leading to improved thermal and mechanical behavior. Recently, Wang and coworkers managed to prepare hyperbranched polyether epoxy structures grafted to the GO layers in order to improve the exfoliation of GO sheets after the introduction into the benzoxazine matrix [35]. Although recent studies have approached the synthesis of dendrimer-based benzoxazine monomers [36,37,38], the covalent growth of benzoxazine monomers starting from dendrimer-functionalized GO has not been previously approached. In this study, starting from the idea that hyperbranched benzoxazine may be synthesized by using poly(amidoamine) dendrimers (PAMAM) as primary amine component in the Mannich condensation, GO surface was chemically modified with PAMAM of different generations in order to directly synthesize numerous BZ rings. Three different generations of PAMAM dendrimers (G0, G1, G2) were employed in order to decorate the surface of GO with numerous benzoxazine structures and their polymerization behavior is investigated. Therefore, this study proposes an original route for the decoration of GO layers with multiple benzoxazine rings and critically describes the exfoliation process that depends on the PAMAM generation and influences the thermal and mechanical properties of the final nanocomposites.

## 2. Materials and Methods 

### 2.1. Materials

The graphene oxide powder (GO) was purchased from NanoInnova Technologies (Toledo, Spain). Poly(amidoamine) dendrimer solutions (ethylenediamine (EDA) core, 20 wt. % in methanol) of generation 0, 1, and 2, namely PAMAM(G0), PAMAM(G1), PAMAM(G2), phenol, paraformaldehyde, sodium hydroxide (NaOH), anhydrous magnesium sulphate (MgSO_4_), chloroacetic acid (ClCH_2_COOH), hydrochloric acid (HCl), ethyl-3-(3-dimethylaminopropyl)-carbodiimide (EDC) and chloroform were supplied by Sigma-Aldrich (Taufkirchen, Germany). N-Hydroxysuccinimide (NHS) was received from Fluka, China.

### 2.2. Synthesis of Carboxylated GO (GO-COOH)

**Figure 1:**
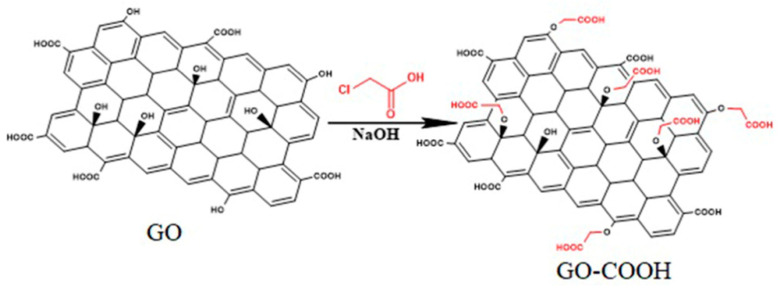
Synthesis of carboxylated graphene oxide.

For the carboxylation of GO, Park’s protocol was adopted [39]. Thus 100 mg of GO were dispersed in 450 mL of distilled water and ultrasonicated for 1 h (5–10 °C, pulse 20, amplitude 60%) to fully exfoliate the graphene layers. Twelve grams of NaOH were slowly added into the yellow-brown suspension maintaining low temperature and ultrasonication conditions for 1 h. Thereafter 12 g ClCH_2_COOH were introduced in order to transform the –OH groups from GO surface into ether bonds with carboxyl end-functionalities (C–O–CH_2_–COOH) as shown in Figure 1. The GO-COOH suspension was neutralized with 37% HCl, filtered, repeatedly washed with distilled water and vacuum dried.

### 2.3. Synthesis of GO-COOH Functionalized with Poly(amidoamine) Dendrimer (GO-PAMAM)

**Figure 2:**
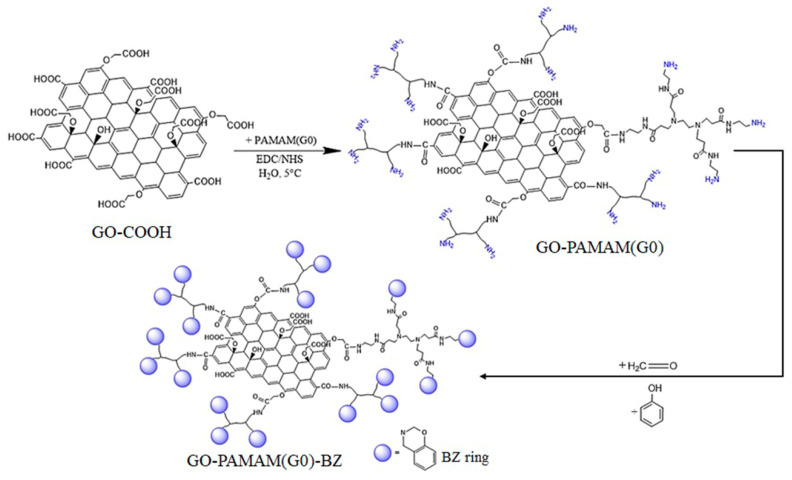
The functionalization route of GO-COOH with multiple benzoxazine rings.

Initially, the PAMAM dendrimers, with EDA core branched from G0 to G2 generation number, were separated from the methanol solution via rotary evaporation and stored as 20 mg/mL aqueous solutions at 2–8 °C. Separately, 3×GO-COOH aqueous suspensions (2 mg/mL) were prepared and ultrasonicated for 30 min (5–10 °C). Thereafter, 300 mg EDC and 200 mg NHS were added in each flask. The role of the EDC/NHS system in this case is to react with the carboxylic groups from GO surface and activate the reaction between –COOH and primary amines from PAMAM structures. Thus, the dendrimer aqueous solution of different generation was added to the activated GO-COOH suspension and the pH of the mixture was adjusted to 6–7 using 37% HCl. The reaction was maintained at 25 °C for 48 h for the conversion of –COOH groups from GO surface into amide bonds (Figure 2). Each GO-PAMAM(G0, G1, G2) product was filtered, thoroughly washed with distilled water, introduced into dialysis sacks (MWCO = 12,000) for 48 h in order to remove any intermediary by-products and freeze-dried.

### 2.4. Synthesis of Benzoxazine-Functionalized GO-PAMAM

For the formation of BZ rings onto the GO surface, each GO-PAMAM of different generation previously synthesized was considered as the amine component in the Mannich condensation reaction with paraformaldehyde and phenol. Therefore, depending on the number of amino surface groups from each PAMAM dendrimer used for GO-COOH functionalization, the necessary amount of reagents was determined. The amine, paraformaldehyde and phenol components were reacted in 1:2:1 molar ratio using chloroform as a solvent and the reaction was maintained under reflux for 6 h at 65 °C. The GO-PAMAM(G0 ÷ 2)-BZ products were purified using 1 M NaOH aqueous solution in order to remove the unreacted phenol and the solvent was eliminated through rotary evaporation. Figure 2 is depicting the synthesis route for the functionalization of GO-COOH with benzoxazine structures using PAMAM(G0). Similar products were obtained when PAMAM(G1) and PAMAM(G2) were employed in the hybrid BZ monomers synthesis exhibiting hyperbranched structures with benzoxazine moieties.

**Figure 3:**
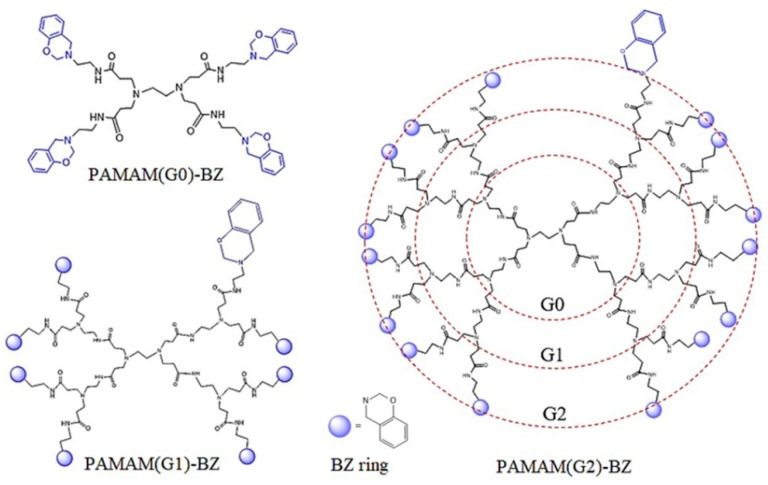
The final structures of benzoxazine (BZ) monomers obtained from poly(amidoamine) dendrimers (PAMAM) dendrimers.

The molecular structure of the intermediary and final products was investigated through FT-IR and ^1^H-NMR to confirm the formation of the benzoxazine rings. The results for synthesized hybrid materials were compared to BZ monomers separately prepared using PAMAM(G0 ÷ 2), paraformaldehyde and phenol according to Lu et al. protocol [36] and their final chemical structure is presented in Figure 3. The solvent was evaporated under vacuum to obtain a yellow product. FT-IR (KBr, cm^−1^): 1220 and 1035 (asymmetric and asymmetric C–O–C stretching), 925 (oxazine ring), 756 (ortho-disubstituted benzene). ^1^H-NMR (600 MHz, DMSO-*d_6_*): δ = 7.07–6.68 (m, 16H, aromatic CH), δ = 4.8 (s, 4H, O–CH_2_–N), δ = 3.9 (s, 4H, Ar–CH_2_–N).

### 2.5. Measurements

Fourier transformed infrared (FT-IR) spectra were registered on Bruker Vertex70 spectrometer (Billerica, MA, USA). The samples were prepared in KBr pellets and 32 scans were recorded at 4 cm^−1^ cm resolution. 

The NMR spectra were recorded on a Bruker Advance III HD spectrometer (Bruker, Rheinstetten, Germany) operating at 600.12 MHz for 1H. For the NMR analysis, a 5 mm multinuclear inverse detection z-gradient probe was used. The analyses were performed using deuterated dimethylsulfoxide as solvent and tetramethylsilane as internal standard. ^1^H–NMR spectra were registered using a standard pulse sequence, as delivered by Bruker, with TopSpin 3.5.6 spectrometer control and processing software.

X-ray photoelectron spectroscopy (XPS) analyses were performed on a Thermo Scientific K-Alpha equipment (Ho Chi Minh, Vietnam), using a monochromatic Al Ka source (1486.6 eV), at a pressure of 2 × 10^−9^ mbar. The binding energy was calibrated by placing the C1s peak at 284.8 eV as internal standard. 

Raman investigation was done using a Renishaw inVia Raman microscope system 473 nm laser excitation, 100× objective and 0.4 mW incident power.

The X-ray diffraction analyses (XRD) were performed on a Panalytical X’PERT MPD X-ray Diffractometer (Malvern Panalytical, Royston, UK), in the range 2θ = 2–50°. An X-ray beam characteristic to Cu Kα radiation was used (λ = 1.5418 Å).

Thermogravimetric analyses (TGA) were performed with a Q500 TA instrument from 30 to 800 °C using nitrogen (gas flow rate of 90 mL/min and 10 °C/min as heating rate). 

Differential scanning calorimetry (DSC) curves were recorded on DSC 402 F 1 equipment from Netzsch (Selb, Germany). The non-isothermal method was used to scan the samples under nitrogen, from 30 to 300 °C using a 5 °C/min heating rate.

Hardness (H) and Young’s modulus (E) were determined using a Nanoindenter G200, Agilent Technologies (Santa Clara, CA, USA). The samples were fixed on sample holder for the NanoVision stage. All indents were performed using a Berkovich diamond tip with a 20-nm radius. The indentations were performed using the Express Test to a Displacement method from the NanoSuit software; for each sample 100 indents at a 50 μm distance from each other at a displacement into the surface of 1000 nm and a poisson ratio of 0.17 were done.

## 3. Results and Discussion

### 3.1. XPS Investigation

**Figure 4:**
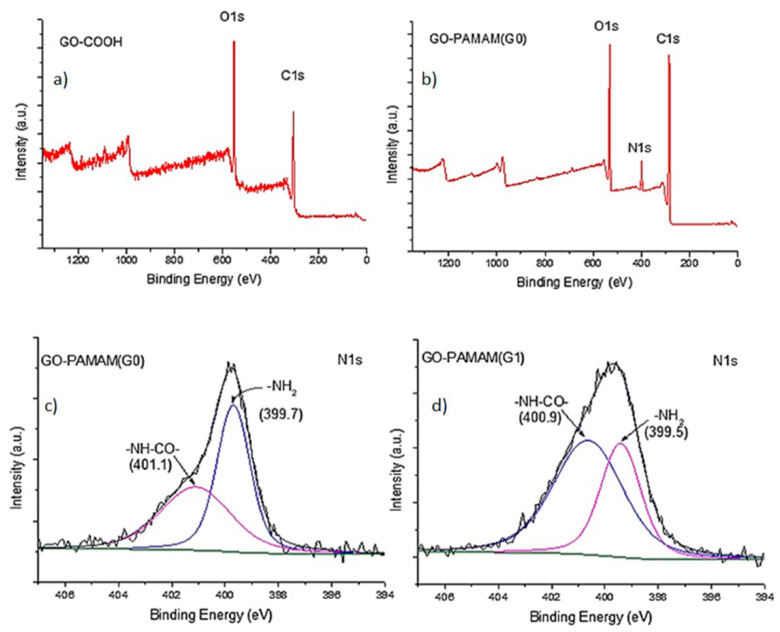
The XPS Survey spectra of GO-COOH before (**a**) and after functionalization (**b**) with PAMAM(G0) and N1s deconvolution spectra of GO-PAMAM(G0) (**c**); GO-PAMAM(G1) (**d**) structures.

Firstly, GO-COOH surface loading with dendrimer structures was investigated through XPS to probe the chemical attachment of hyperbranched poly(amidoamines). As observed in Figure 4 the XPS Survey spectrum of the carboxylated GO shows binding energies at only 284.8 eV and 532 eV, corresponding to C1s and O1s peaks [40]. New signal occurs in the XPS Survey spectra of GO-PAMAM structures corresponding to N1s peak from the nitrogen containing dendrimers. As a result, the atomic composition for each material is modified with increasing the PAMAM generation (Table 1). Moreover, the attachment of primary amine functionalities was revealed by N1s XPS deconvolution spectra showing the presence of –NH_2_ (~399.7 eV) and amide bonds (~401 eV) indicating that the amino functionalization has indeed taken place at the surface of graphene oxide [41].

### 3.2. FT-IR Analyses

**Figure 5:**
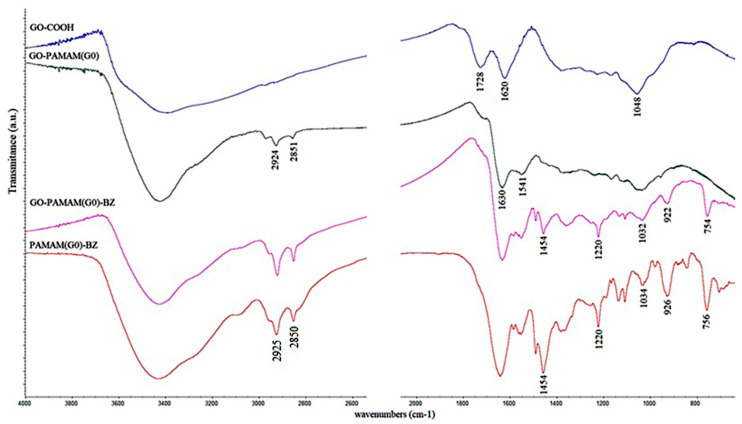
FT-IR spectra of hybrid monomers GO-PAMAM(G0)-BZ.

The reaction progress from GO-COOH to BZ-functionalized GO-PAMAM structures was investigated through FT-IR spectrometry (Figure 5). Similar spectra were registered for PAMAM(G1, G2)-based structures. The FT-IR spectrum for the raw material GO-COOH showed the presence of the carboxyl groups (1728 cm^−1^) [42], broad -OH signal (~3300 cm^−1^), C=C bonds from the planar structure of graphene (1620 cm^−1^) and some residual C–O–C groups (1048 cm^−1^) characteristic to GO structure [43,44]. The interaction of GO-COOH with PAMAM dendrimers led to new signals appearance in the FT-IR spectra of each GO-PAMAM synthesized. The bands located at ~1630 cm^−1^ and ~1540 cm^−1^ are assigned to the O=C–NH stretching vibrations from PAMAM structures. Additionally, the presence of the signals from ~2925 cm^−1^ and ~2850 cm^−1^ corresponding to –CH_2_– groups from the ethylene core of the dendrimers is confirming that amidoamines groups were covalently grafted to GO-COOH [45]. The formation of the hybrid GO-PAMAM-BZ monomers was proven through FT-IR investigation for each dendrimer generation employed in the synthesis by comparing the final products with BZ monomers obtained from PAMAM dendrimers as amine source for BZ synthesis. Therefore, as observed from the FT-IR spectra, the characteristic signals of the benzoxazine structure were pointed out through the absorbance signals from ~1030 cm^−1^ and ~1220 cm^−1^ corresponding to ether bond from the oxazine ring (C–O–C symmetric and asymmetric stretching mode) [36]. In addition, the ~920 cm^−1^ and ~750 cm^−1^ peaks assigned to the oxazine cycle and ortho-disubstituted benzene were revealed. The formation of the oxazine ring grafted from GO-COOH was also demonstrated through ^1^H-NMR analyses.

### 3.3. ^1^H-NMR Results

**Figure 6:**
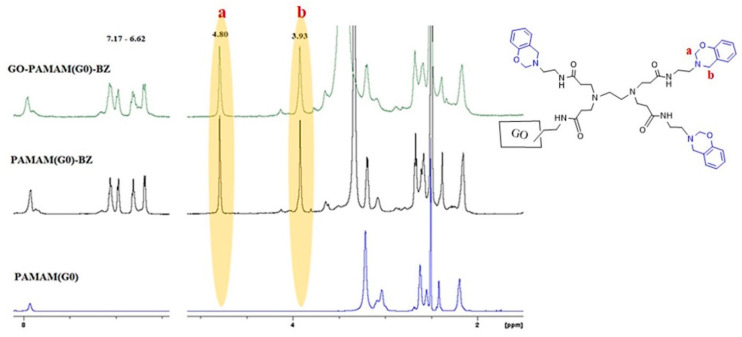
^1^H-NMR spectra for PAMAM(G0), PAMAM(G0)-BZ and GO-PAMAM(G0)-BZ.

**Figure 7:**
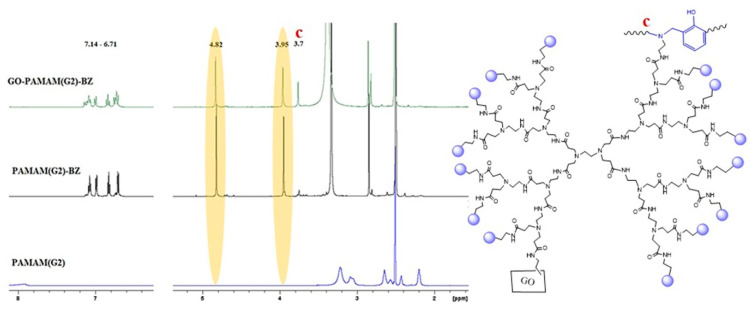
^1^H-NMR spectra for PAMAM(G2), PAMAM(G2)-BZ and GO-PAMAM(G2)-BZ.

Figure 6 shows the ^1^H-NMR spectra of the PAMAM dendrimers and their corresponding benzoxazine monomers and final GO-PAMAM-BZ structures for the case of PAMAM(G0). Similar spectra were registered for the structures based on PAMAM(G1). The presence of the protons from –N–CH_2_–Ar– (3.9 ppm) (b) and O–CH_2_–N– (4.8 ppm) (a) units in the ^1^H-NMR spectra of each GO-PAMAM-BZ compound demonstrates that pendant benzoxazine structures are formed onto GO-PAMAM [36]. The presence of the aromatic rings is noticed at 7.1–6.6 ppm. In case of GO-PAMAM(G2)-BZ an additional signal was observed at 3.7 ppm (c) corresponding to Mannich type structures, indicating that ring opening polymerization of BZ rings has occurred during the synthesis (Figure 7) [46].

### 3.4. Raman Spectrometry

**Figure 8:**
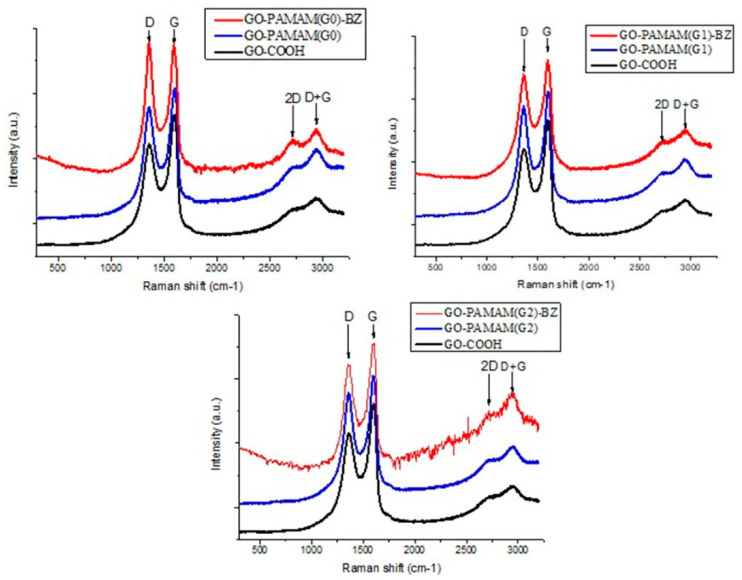
The Raman spectra of the raw material GO-COOH and hybrid monomers GO-PAMAM-BZ.

The arrangement of the GO layers during the functionalization with hyperbranched dendrimers and benzoxazine structures was evaluated through Raman spectrometry. This non-destructive technique provides useful information regarding the extent of functionalization, the formation of defects and the number of GO layers. As shown in Figure 8, the Raman spectrum of the investigated GO derivatives exhibits two distinguishable peaks at ~1360 cm^−1^ (D band) and ~1590 cm^−1^ (G band). The D band is caused by the existence of sp^3^ hybridized C atoms considered as defects in the sp^2^ planar sheet of graphene subsequently functionalized. The G band is characteristic to all sp^2^ hybridized carbon nanomaterials and arises as a consequence of the stretching vibrations of C-C bond from the planar structure of graphene. Another important Raman signal is the 2D peak (~2700 cm^−1^) that characterizes the arrangement and the number of layers for the graphene-like materials.

The broadening of D and G bands after functionalization and the increase of the ratio between the intensity of D and G band (I_D_/I_G_) are strong indicators of structural changes in the graphene layers during the functionalization suggesting important information regarding the content of the sp^2^ hybridized carbon atoms within the materials [47,48] (Table 2). In case of GO-PAMAM(G0 ÷ 2) it was clearly observed that the ID/IG ratio has increased as a result of more sp^3^ hybridized C atoms located within the GO structure, due to the reaction conditions with PAMAM. Further, when GO-PAMAM(G0) reacts to form BZ rings bonded to the PAMAM moieties, a higher increase of I_D_/I_G_ ratio occurs probably because of more defects of sp^3^ C atoms appeared within the GO structure. However, this is not valid for GO-PAMAM(G1, G2)-BZ structures, for which the ID/IG ratio decreases again since the high number of BZ rings formed may interact with the graphene lattice through the rearrangement of π-π stacking, thus contributing to a smaller number of defects. Moreover, the shape of 2D band from the Raman spectra (Figure 8) may explain a lot about the processes developed during the formation of GO-PAMAM(G0-2)-BZ structures. Thus, the sharpness of 2D band increased as the reaction goes on from GO-COOH to GO-PAMAM and finally to GO-PAMAM-BZ. This means that the assembly of graphene oxide layers is affected through the formation of intercalated and even exfoliated structures because of PAMAM chains and mostly due to BZ and PBZ structures finally achieved.

It can be noticed that the 2D band is sharper for PAMAM(G0)-based structures which means that in this case only a few layers of GO are still forming the stacking layers, the other layers being penetrated by the PAMAM and BZ voluminous structures. For PAMAM(G1, G2)-based structures, this is slightly different since the same tendency of GO layers disintegrations occurs, but this is coupled with a partially process of polymerization of BZ rings. 

**Figure 9:**
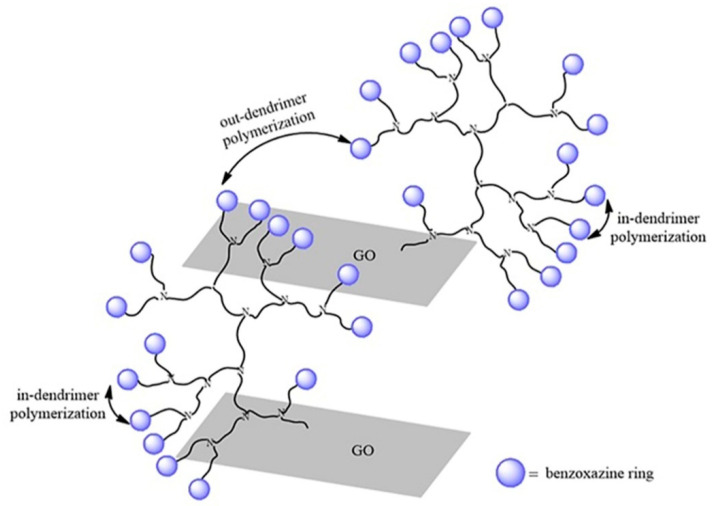
Polymerization routes for hybrid GO-PAMAM functionalized with benzoxazine rings.

The propagation of the polybenzoxazine formation is more likely to take place between benzoxazine rings located closer one to another on the same dendrimer branch (in-dendrimer polymerization) producing intercalated structures due to high density of intra- and intermolecular hydrogen bonding and forming a rigid network structure. Out-dendrimer polymerization occurring between benzoxazine rings from different dendrimer branches leads to GO interlayer disaggregation and the final exfoliated or intercalated structure of the hybrid nanocomposite is determined by the polymerization occurrence (Figure 9). The results are in good agreement with ^1^H-NMR analyses which showed that polybenzoxazine structures are formed as well apart from GO-PAMAM(G2)-BZ.

### 3.5. XRD Tests

**Figure 10:**
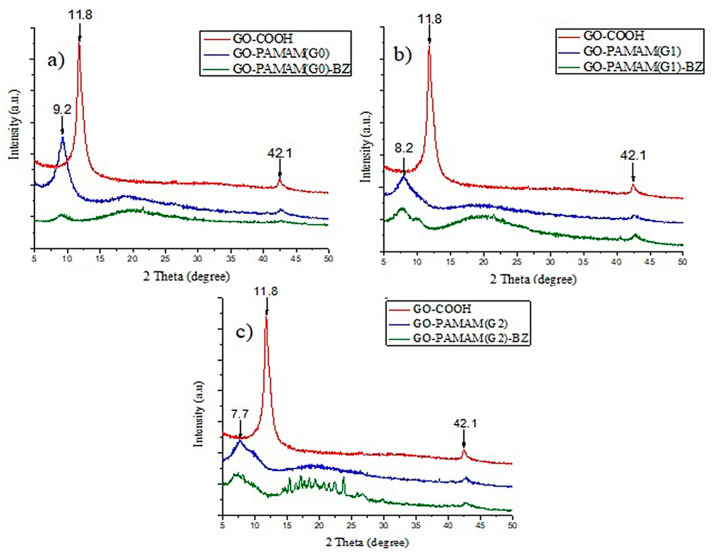
XRD curves for the investigated materials: (**a**) GO-PAMAM(G0)-BZ, (**b**) GO-PAMAM(G1)-BZ, (**c**) GO-PAMAM(G2)-BZ.

Figure 10 shows the XRD curves for the investigated materials. The XRD pattern of GO-COOH shows a sharp peak at 2θ = 11.85° corresponding to (001) graphene oxide and another less intense peak at 2θ = 42.1° common to graphene materials [49]. After the functionalization with PAMAM, the (001) peak position is shifted to lower degrees and the intensity of the signals is significantly decreased as a result of GO layers disaggregation caused by the chemical introduction of hyperbranched structures between GO sheets. After synthesis of GO-PAMAM(G0), the intensity of 2θ signal from 9.19° is significantly decreased which means that intercalated structures are formed through the dispersion of GO layers. At higher degree of benzoxazine functionalization, the interplanar spacing is increased due to separation of GO layers. Intercalated structures are obtained in case of GO-PAMAM(G1)-BZ and clearly more exfoliated structures in case of GO-PAMAM(G2)-BZ (Table 3), probably due to the effect of BZ polymerization showed by ^1^H-NMR data and confirmed from Raman spectra.

### 3.6. TGA Data

**Figure 11:**
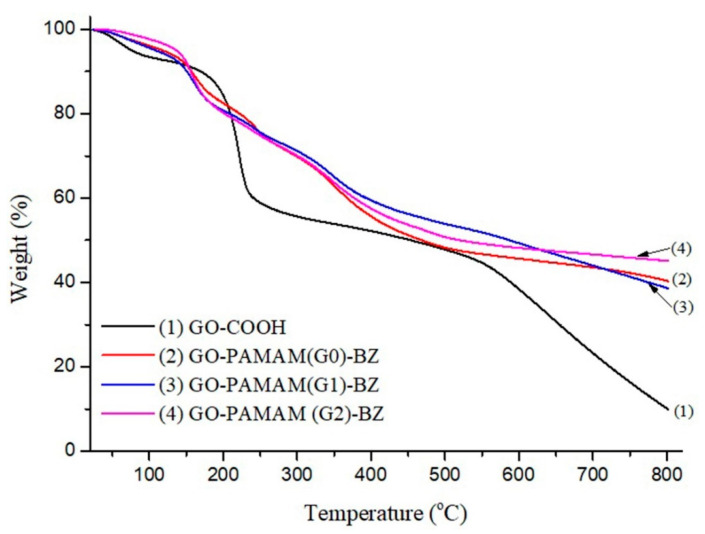
TGA curves for GO-COOH and final benzoxazine functionalized products.

TGA investigation was employed in order to evaluate the thermal stability of the hybrid GO-based materials. As displayed in Figure 11, the GO-COOH thermogram indicates two stages of decomposition: Firstly, the thermal evaporation of the adsorbed moisture from the hydrophilic GO-COOH surface takes place below 100 °C and secondly, the thermal degradation of the oxidized functionalities by releasing CO and CO_2_ that occurs below 200 °C [30]. A supplementary degradation stage at ~240 °C is observed in case of GO-PAMAM(G0 ÷ 2)-BZ attributed to the thermal decomposition of the poly(amidoamine) chains [50]. The thermal stability of GO-PAMAM(G0)-BZ is noticeably increased compared to the raw and intermediary materials (Table 4) due to formation of aromatic oxazine rings onto the GO-PAMAM surface. As expected, in case of GO-PAMAM(G2)-BZ the thermostability is much higher caused by the higher density of H-bonding resulted during the polymerization of the benzoxazine structures, the polybenzoxazine chains being already confirmed by ^1^H-NMR spectra. However, a slight thermal stability decrease is observed in case of GO-PAMAM(G1)-BZ compared to GO-PAMAM(G0)-BZ probably due to formation of less benzoxazine rings and the existence of some free poly(amidoamine) functionalities.

### 3.7. DSC Results

**Figure 12:**
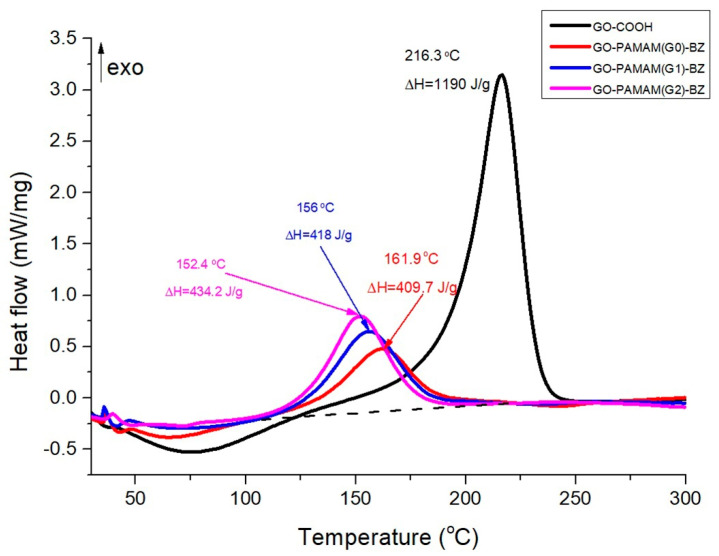
DSC curves for GO-COOH and final benzoxazine functionalized products.

Figure 12 shows the DSC curves of GO-COOH and the final GO-PAMAM-BZ materials. The DSC curve of GO-COOH presents a sharp peak at 216 °C assigned to the release of CO_2_ and CO from the decomposition of oxygen containing functional groups, in good correlation with the results reported by Qiu et al. [51]. This process is also evidenced in TGA curves (Figure 11) since a significantly mass loss occurred between 190 °C and 220 °C. 

The DSC peak at 216 °C is no longer observed on the DSC curves for GO-PAMAM-BZ products which is a strong proof that the functionalization with PAMAM molecules really occurred at the carboxylic groups from the GO surface. Moreover, a sharp peak is noticed for all the GO-PAMAM-BZ samples at a temperature value depending on the generation of PAMAM employed, being attributed to the polymerization of the BZ rings synthesized on the GO-PAMAM surface. Thus, for GO-PAMAM(G0)-BZ, this peak is located at 161.9 °C and it is shifted to lower temperature values as the generation increased. This may be explained by the auto-catalytic effect of BZ rings which being located at lower distance between them as the generation of PAMAM increases will be easier available for polymerization, either through in-dendrimer polymerization or out-dendrimer polymerization (Figure 9). Therefore, in the case of GO-PAMAM(G2)-BZ, the polymerization peak is located at 152.4 °C which is a very good improvement in comparison with the high temperature usually required for benzoxazine polymerization (180–220 °C) [52] and PAMAM-BZ monomers obtained by Lu et al. [36]. This performance may open new fields for use of polybenzoxazines as the BZ polymerization temperature is not a barrier anymore due to its high value.

Polymerization of BZ rings achieved onto the GO-PAMAM surface also reveals a strong dependence of the polymerization heat on the PAMAM generation. This was obviously expected as the highest generation of PAMAM employed (G2) will allow the formation of the highest number of BZ units and therefore a higher value of polymerization heat (434.2 J/g) is obtained. However, this value is not considerably high and may be proper removed during an industrial process which is worthy to consider if the general properties and especially the mechanical ones will be higher than for classical benzoxazines. The value of the polymerization heat for GO-PAMAM(G2)-BZ is limited since a partial polymerization process of BZ units formed through synthesis reaction already occurred as it was revealed by ^1^H-NMR spectra.

### 3.8. Nanoindentation 

**Figure 13:**
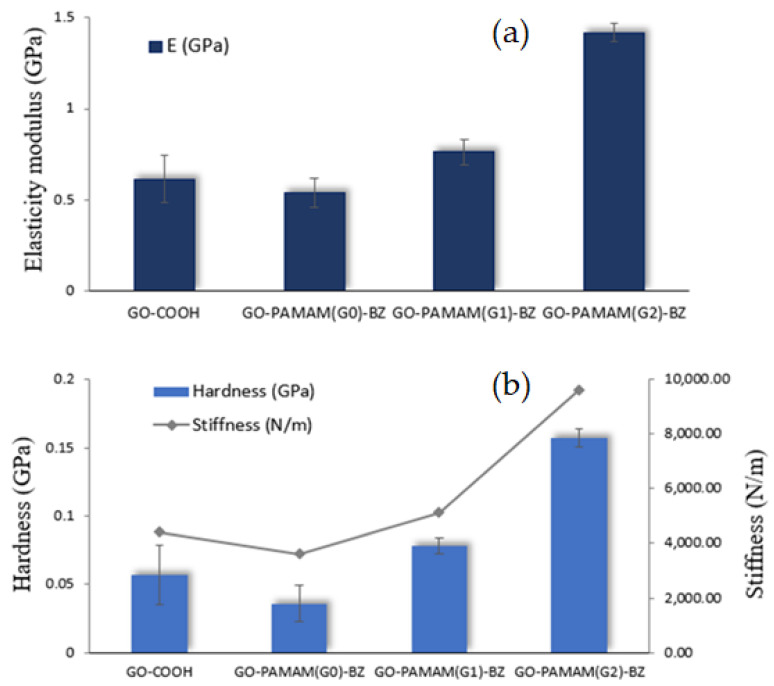
Nanomechanical properties: (**a**) Young’s modulus and (**b**) stiffness and hardness of the raw material (GO-COOH) and hybrid materials.

The nanomechanical properties of GO-PAMAM(G0 ÷ 2)-BZ were evaluated by nanoindentation technique and the average values of Young’s modulus, stiffness, and hardness are presented in Figure 13. The degree of GO dispersion and the homogeneity of the analyzed hybrid samples highly influence the nanomechanical properties of the final nanocomposites [53]. The raw material GO-COOH shows the elasticity modulus (E) of 0.614 GPa, hardness (H) of 0.057 GPa and stiffness (S) of 4416 N/m (Table 5). It is noticed that the mechanical properties of hybrid materials are directly influenced by the generation of dendrimers used in the GO functionalization step. Therefore, a minor decrease of mechanical properties compared to GO-COOH was observed for GO-PAMAM(G0)-BZ in terms of Young’s modulus and stiffness as a consequence of introduction of organic functionalities which makes the structure of the material less compact, thus contributing to more voids within the material. By increasing the PAMAM generation, the number of benzoxazine rings is increased and minor mechanical properties improvement may be observed in case of GO-PAMAM(G1)-BZ. However, the most relevant results are obtained in case of GO-PAMAM(G2)-BZ showing a significant increase of all the mechanical properties studied probably due to the highly crosslinked network of polybenzoxazine, formed through the numerous BZ rings which are suitable for polymerization being located at less distance.

## 4. Conclusions

New nanomaterials based on benzoxazine (BZ)-functionalized graphene oxide (GO) -poly(amidoamines) (PAMAM) structures were synthesized to gain a lot of BZ rings on the materials surface, capable to form a crosslinked network by polymerization.

The XPS curves, FTIR and ^1^H-NMR spectra confirmed BZ rings formation at the amine functional groups of PAMAM. However, this process depends on the number of amino functional groups from PAMAM, meaning that the generation of PAMAM has a significant influence on the final morphology of GO-PAMAM-BZ structures. Thus, by employing PAMAM(G2) with a higher number of amino groups, more BZ rings are formed and these will further react to form polybenzoxazine chains which are directly detected from ^1^H-NMR spectra and indirectly from XRD, TGA and nanoindentation. Therefore, the mechanical properties of GO-PAMAM(G2)-BZ are significantly higher than for the other two PAMAM generations (G0, G1)-based materials. 

The formation of BZ units at the surface of PAMAM amino functionalities exhibits also an important influence to the intercalation process of GO layers since the XRD data showed an increase of the basal distance between the GO layers probably due to the partial polymerization of BZ-functionalized PAMAM chains within the GO layers. This fact is more obvious in the case of GO-PAMAM(G0)-BZ by appearance of sharper 2D band in the Raman spectrum which means a few layers of graphene oxide which are still stacked in assemblies. This effect is less noticeably for GO-PAMAM(G2)-BZ from Raman spectra, but XRD data show in this case the highest degree of intercalation among all the generations of PAMAM-based materials.

The polymerization process of BZ units achieved by synthesis onto GO-PAMAM surface occurs at a temperature which strongly depends on the generation of PAMAM employed. As the generation of PAMAM increased, a less distance between the BZ units is obtained, that will favor the polymerization process and therefore the polymerization occurs at lower temperatures. As a consequence, for all the GO-PAMAM-BZ products, the polymerization temperature is significantly less than for classical benzoxazines which may open industrial applications for these structures considering that for most of the BZ used in different applications, the high polymerization temperature is a significant disadvantage. Due to the promising results obtained in this study, the functionalization of GO layers with benzoxazine monomers using dendrimers of higher generations needs to be performed for further investigation of the thermal and mechanical properties of the GO-PAAMAM-BZ nanocomposites. 

## Figures and Tables

**Table 1:** Atomic composition of GO-COOH before and after PAMAM functionalization.

Sample	C (%)	O (%)	N (%)
GO-COOH	67	33	0
GO-PAMAM(G0)	76	20	4
GO-PAMAM(G1)	72	20	8
GO-PAMAM(G2)	71	20	9

**Table 2:** The I_D_/I_G_ ratio resulted from the Raman spectra of investigated materials.

Sample	I_D_/I_G_ (473nm)
GO-COOH	0.78
GO-PAMAM(G0)	0.85
GO-PAMAM(G1)	0.86
GO-PAMAM(G2)	0.86
GO-PAMAM(G0)-BZ	1.02
GO-PAMAM(G1)-BZ	0.88
GO-PAMAM(G2)-BZ	0.83

**Table 3:** Structural parameters obtained from XRD patterns.

Sample	2θ Position (°)	d-spacing (Å)
GO-COOH	11.85	7.46
GO-PAMAM(G0)	9.24	9.61
GO-PAMAM(G1)	8.22	10.74
GO-PAMAM(G2)	7.72	11.44
GO-PAMAM(G0)-BZ	9.19	9.65
GO-PAMAM(G1)-BZ	7.7	11.46
GO-PAMAM(G2)-BZ	7.01	12.61

**Table 4:** The TGA data for GO-COOH and final products.

Sample	Weight Loss, % (25–800 °C)	Td_3%,_ °C	Td_5%,_ °C
GO-COOH	90	58.8	76.7
GO-PAMAM(G0)-BZ	59.7	84.9	118.6
GO-PAMAM(G1)-BZ	61.4	80.8	109.3
GO-PAMAM(G2)-BZ	54.9	113.1	136.8

**Table 5:** Mechanical properties of GO-COOH and GO-PAMAM(G0÷2)-BZ materials obtained by nanoindentation.

Sample	E (GPa)	Hardness (GPa)	Stiffness (N/m)
GO-COOH	0.614	0.057	4416
GO-PAMAM(G0)-BZ	0.536	0.036	3617
GO-PAMAM(G1)-BZ	0.763	0.078	5117
GO-PAMAM(G2)-BZ	1.418	0.157	9621
